# Sex Differences in Social Cognition and Association of Social Cognition and Neurocognition in Early Course Schizophrenia

**DOI:** 10.3389/fpsyg.2022.867468

**Published:** 2022-04-15

**Authors:** Ryotaro Kubota, Ryo Okubo, Satoru Ikezawa, Makoto Matsui, Leona Adachi, Ayumu Wada, Chinatsu Fujimaki, Yuji Yamada, Koji Saeki, Chika Sumiyoshi, Akiko Kikuchi, Yoshie Omachi, Kazuyoshi Takeda, Ryota Hashimoto, Tomiki Sumiyoshi, Naoki Yoshimura

**Affiliations:** ^1^Department of Psychiatry, National Center of Neurology and Psychiatry Hospital, Tokyo, Japan; ^2^Clinical Research and Education Promotion Division, National Center of Neurology and Psychiatry, Tokyo, Japan; ^3^Endowed Institute for Empowering Gifted Minds, Graduate School of Arts and Sciences, The University of Tokyo, Tokyo, Japan; ^4^Department of Preventive Intervention for Psychiatric Disorders, National Institute of Mental Health, National Center of Neurology and Psychiatry, Tokyo, Japan; ^5^Faculty of Human Development and Culture, Fukushima University, Fukushima, Japan; ^6^Department of Pathology of Mental Diseases, National Institute of Mental Health, National Center of Neurology and Psychiatry, Tokyo, Japan; ^7^Department of Community Mental Health and Law, National Institute of Mental Health, National Center of Neurology and Psychiatry, Tokyo, Japan

**Keywords:** schizophrenia, social cognition, neurocognition, theory of mind (ToM), hostility bias, sex difference

## Abstract

**Background:**

Both impairment and sex differences in social cognition and neurocognition have been documented in schizophrenia. However, whether sex differences exist in the association between social cognition and neurocognition are not known. We aimed to investigate the contribution of areas of neurocognition to theory of mind (ToM) and hostility bias, representing social cognition, according to sex in early course schizophrenia.

**Methods:**

In this cross-sectional study, we assessed neurocognition using the Japanese version of the Brief Assessment of Cognition in Schizophrenia (BACS) and assessed the ToM and hostility bias subdomains of social cognition using the Social Cognition Screening Questionnaire (SCSQ) in 131 participants (65 female, 66 male) diagnosed with schizophrenia within 5 years of onset. Sex differences were analyzed using *t*-tests. The associations of neurocognitive subdomains with ToM and hostility bias according to sex were analyzed using multiple regression analysis. Results were adjusted by age, estimated premorbid intelligence quotient, and symptomatology.

**Results:**

No sex differences were found in ToM (*p* = 0.071) or hostility bias (*p* = 0.057). Higher verbal fluency was significantly associated with higher ToM in females (*p* < 0.01), whereas higher executive function was significantly associated with higher ToM in males (*p* < 0.05). Higher verbal fluency was significantly associated with lower hostility bias in females (*p* < 0.05), whereas neurocognition and hostility bias were not significantly associated in males.

**Conclusion:**

The results suggest that neurocognition associated with social cognition differ according to sex. These differences should be considered for more effective treatment of social cognition.

## Introduction

Kraepelin (1919) noted that there is a sex/gender difference in schizophrenia and wrote: “The male sex appears in general to suffer somewhat more frequently from the dementia praecox than the females.” For the past decade, “sex” has been used to refer to biological variables, and “gender” has been used for psychosocial variables (Mendrek and Mancini-Marie, 2016). In recent years, considerable attention has been given to the treatment of social cognition in schizophrenia, including social cognitive training programs and pharmacotherapies, which take into account biological aspects (Javed and Charles, 2018). We use the word “sex” instead of “gender” to indicate that we are discussing the biological aspect in schizophrenia through an assessment of social cognition.

Social cognition is defined as “the mental operations that underlie social interactions, including perceiving, interpreting, and generating responses to the intentions, dispositions, and behaviors of others” (Green et al., 2008). This multidimensional construct is composed of the subdomains of emotional processing, theory of mind (ToM), social perception, and attributional bias (Pinkham et al., 2014). In patients with schizophrenia, impairment of social cognition is considered one of the greatest obstacles to social participation, including that involving interpersonal relationships, education, and employment (Green et al., 2012; Kubota et al., 2021). Over the past decade, considerable attention has been given to the development of new therapies targeting social cognitive deficits as a means to improve functional outcomes in schizophrenia (Green et al., 2019). As some patients in the early course schizophrenia have been reported to respond to both pharmacological and psychosocial treatments (Robinson et al., 1999; Catalan et al., 2018), the optimal time to intervene to prevent future decline might be early in its course (Crumlish et al., 2009; Catalan et al., 2018).

Sex differences have been shown to affect not only functional outcomes (Seeman, 2019), but also social cognition in schizophrenia, and therefore any intervention in early course schizophrenia to improve social cognition should account for these sex differences (Chen et al., 2021). Research on sex differences in social cognition has been conducted to improve functional outcomes. According to previous studies (Buck et al., 2016; Roberts et al., 2016), impairment of social cognition in schizophrenia has a two-factor structure, social cognitive skills including ToM and attributional bias including hostility bias (Buck et al., 2016; Roberts et al., 2016). In terms of sex differences in ToM, there are mixed results indicating either no sex differences or better ToM in females (Abu-Akel and Bo, 2013; Ayesa-Arriola et al., 2014; Navarra-Ventura et al., 2018, 2021; Riecher-Rossler et al., 2018). These conflicting results might be due to differences in disease duration between the studies. A meta-analysis has not yet been carried out, and no conclusion about sex differences in ToM has been reached. To our knowledge, hostility bias has not yet been investigated separately for females and males, so there is a need to investigate potential sex differences.

Treatment of social cognitive functioning should take into account neurocognitive functioning (Deckler et al., 2018; Thibaudeau et al., 2020), and combining the treatment of neurocognitive impairment and social cognitive impairment may improve functional outcomes (Deckler et al., 2018). A number of studies have examined the association between ToM and neurocognition, with a meta-analysis showing that neurocognition such as attention, working memory, episodic memory, speed of processing, language, visuospatial/problem solving and executive functions influence ToM to the same extent, but with no specific neurocognitive area having a pronounced effect on ToM (Thibaudeau et al., 2020). It is possible that several areas influence ToM to the same extent, because that meta-analysis did not account for sex differences. Given that sex differences affect both social cognition and neurocognition (Longenecker et al., 2010; Chen et al., 2021), sex differences need to be taken into account when examining the association between ToM and neurocognition. However, to our knowledge, no studies have done this to date. As for hostility bias, although studies have examined its association with neurocognition (Donohoe et al., 2008; Buck et al., 2017, 2020; Lo and Siu, 2017), the analyses were not conducted separately for female and male subjects. Therefore, the association between hostility bias and neurocognition needs to be examined by sex.

In efforts to facilitate more individualized assessment and treatment of social cognition in patients with early course schizophrenia, this study sought to clarify the association between neurocognitive and social cognitive subdomains in early course schizophrenia by sex. Specifically, first we examined sex differences in ToM and hostility bias in early course schizophrenia and then investigated the contribution of neurocognition to ToM and hostility bias by sex.

## Methods and Analysis

### Participants

In this cross-sectional study, we recruited 131 patients with schizophrenia (65 female, 66 male) within 5 years of onset who visited the Early Detection and Intervention Center for Schizophrenia (EDICS) at the National Institute of Neurology and Psychiatry between December 2013 and December 2020. Schizophrenia was diagnosed according to the Diagnostic and Statistical Manual of Mental Disorders-5 (DSM-5). Patients were excluded if they had concomitant disease with a marked effect on cognition such as dementia, their physician judged that they would potentially be disadvantaged by participating (e.g., relapse, excitement), or the principal investigator deemed their participation inappropriate. Following approval of the study design by the Institutional Review Board of the National Center of Neurology and Psychiatry (Approval No. A2018-139), written informed consent was obtained from all participants. When the participant was a minor (i.e., < 20 years of age), written consent was obtained from a parent or guardian, with additional written assent obtained from patients aged 16–19 years.

### Measures

The Social Cognition Screening Questionnaire (SCSQ) was used to assess social cognition. This quick and objectively scored instrument was designed to help busy clinicians practicing in real-world clinical settings to conduct a standard assessment of treatment (Roberts et al., 2016). It consists of five domains: verbal memory, schematic inference, ToM, metacognition, and hostility bias. For each domain except for hostility bias, higher scores indicate higher performance. We used the scores for ToM and hostility bias to represent social cognition in this study. The reliability and validity of the Japanese version of the SCSQ have been verified. The validity of ToM and hostility bias domains were examined by investigating their correlation with the Hinting task, the Ambiguous Intention Hostility Questionnaire (AIHQ), and the Social Functioning Scale, respectively (Roberts et al., 2011; Kanie et al., 2014).

The Japanese version of the Brief Assessment of Cognition in Schizophrenia (BACS) was used to assess neurocognition. The BACS was developed to assess cognitive impairment in schizophrenia and is currently widely used to assess psychiatric disorders. It consists of 6 domains: verbal memory, working memory, motor speed, attention, verbal fluency, and executive functions. Higher scores indicate higher performance. The reliability and validity of the Japanese version have been verified (Keefe et al., 2004; Kaneda et al., 2007).

The Positive and Negative Syndrome Scale (PANSS) was used to assess overall psychiatric symptoms in schizophrenia during an interview. This scale includes a positive symptom subscale, negative symptom subscale, and general psychopathology subscale. Each item is rated from 1 (*absent*) to 7 (*extreme*). The Japanese version has been validated (Kay et al., 1987; Igarashi et al., 1998; Hashimoto et al., 2020).

The 25-item short version of the Japanese Adult Reading Test (JART25) was used to assess estimated pre-morbid IQ. The JART is the Japanese version of the National Adult Reading Test (Nelson and O’Connell, 1978). The JART25 tests the reading of 25 kanji characters (Matsuoka et al., 2006).

The Global Assessment of Functioning (GAF) was administered to provide a global assessment of the patient’s condition, ranging from good mental health to severe psychopathology. It is a general rather than diagnostic-specific scoring system. The 100-point GAF scale is divided into 10-point intervals (sections) (American Psychiatric Association [APA], 1994).

Lastly, factors affecting neurocognition and social cognition were examined, including age, disease duration, years of education, estimated pre-morbid IQ, and psychiatric symptoms (Pinkham et al., 2017; Chen et al., 2021; Navarra-Ventura et al., 2021).

### Statistical Analysis

Variables are described using means and standard deviations (*SD*). Results for the social cognitive subdomains, neurocognitive subdomains, psychiatric symptoms, and estimated pre-morbid IQ were compared between the female and male participants. Continuous variables were compared using standardized differences and *t*-tests.

Multivariable regression models were used to examine the association between each neurocognitive subdomain and each social cognitive subdomain in female and male participants. Multivariate analyses were performed with psychiatric symptoms, age, estimated pre-morbid IQ, and neurocognition as independent variables. Disease duration and years of education were excluded from the multiple regression analyses because the participants were within 5 years of onset and because estimated pre-morbid IQ and years of education overlapped. Results were considered statistically significant at *p* < 0.05. All statistical analyses were performed using IBM SPSS Statistics version 26.

## Results

### Sociodemographic Variables

Means and standard deviations for demographic variables, clinical variables, and PANSS scores are shown in Table 1. There were no sex differences in factors known to affect neurocognition and social cognition such as age, disease duration, years of education, estimated pre-morbid IQ and psychiatric symptoms.

**TABLE 1:** Sociodemographic variables and clinical data for participants with early course schizophrenia.

		Female (*n* = 65) mean (*SD*)	Male (*n* = 66) mean (*SD*)	*P*	Standardized difference
Age (years)		25.9 (8.2)	25.6 (8.1)	0.849	0.037
Duration (years)		1.46 (1.3)	1.61 (1.3)	0.524	−0.115
Education (years)		13.5 (2.3)	13.9 (2.6)	0.313	−0.163
JART25		98.2 (12.0)	100.6 (11.3)	0.238	−0.206
PANSS					
	Negative	17.9 (6.3)	18.2 (5.0)	0.747	−0.053
	Positive	15.4 (5.6)	14.6 (4.5)	0.394	0.157
	General	36.5 (8.4)	35.0 (6.5)	0.272	0.200
GAF		48.9 (11.3)	49.4 (10.0)	0.807	−0.047

*JART, Japanese Adult Reading Test; PANSS, Positive and Negative Syndrome Scale.*

### Neurocognitive and Social Cognitive Tasks

Results of the BACS and SCSQ are shown in Table 2. Males had significantly higher scores than females for executive function and working memory, but there was no significant sex difference for ToM or hostility bias.

**TABLE 2:** Comparison of cognitive task results between male and female participants.

Cognitive tasks		Female mean (*SD*)	Male mean (*SD*)	*P*
		*N* = 65	*N* = 66	
BACS				
	Verbal memory	41.4 (13.7)	42.1 (11.6)	0.766
	Working memory	17.9 (4.6)	20.0 (4.2)	***0.007***
	Motor speed	64.9 (16.0)	63.3 (16.4)	0.572
	Verbal fluency	38.8 (11.9)	39.0 (10.4)	0.925
	Attention	53.1 (13.0)	53.5 (11.1)	0.853
	Executive function	15.9 (4.3)	17.5 (2.7)	***0.013***
SCSQ				
	ToM	6.68 (2.5)	7.32 (1.5)	0.071
	Hostility	1.66 (1.3)	1.29 (0.9)	0.057

*BACS, Brief Assessment of Cognition in Schizophrenia; SCSQ, Social Cognition Screening Questionnaire. Bold and italic values means statistically significant result.*

### Association Between Neurocognition and Social Cognition

As shown in Table 3, the association between neurocognition and social cognition was assessed after adjusting for age, JART25, and PANSS scores. The results showed statistically significant associations between some of the indicators and ToM and hostility bias, independent of other variables. Higher verbal fluency was significantly associated with higher ToM in females (*p* < 0.01) but not in males (*p* = 0.74), whereas higher executive function was significantly associated with higher ToM in males (*p* < 0.05) but not in females (*p* = 0.92). Higher verbal fluency was significantly associated with lower hostility bias in females (*p* < 0.05) but not in males (*p* = 0.68). None of the neurocognitive subdomains were significantly associated with hostility bias in males. For psychiatric symptoms as measured by the PANSS, none of its three domains (Positive scale, Negative scale, and General Psychopathology scale) was associated with ToM or hostility bias. In all regression models, multicollinearity between predictors was checked. Variance inflation factors ranged from 1.141 to 3.460, all of which fell below the threshold of 10 for adverse multicollinearity.

**TABLE 3:** Association between neurocognitive performance and symptoms with ToM and hostility bias (multivariate analysis).

		ToM						Hostility				
		**Female**			**Male**			**Female**		**Male**		
		**β**	**(95%Cl)**		**β**	**(95%Cl)**		**β**	**(95%Cl)**		**β**	**(95%Cl)**	
BACS													
	Verbal memory	−0.064	−0.082	0.059	0.047	−0.033	0.045	−0.128	−0.054	0.029	0.062	−0.019	0.028
	Working memory	0.085	−0.137	0.228	0.080	−0.081	0.137	0.005	−0.106	0.109	−0.091	−0.085	0.046
	Motor speed	0.066	−0.034	0.054	−0.034	−0.031	0.025	−0.100	−0.034	0.018	0.126	−0.010	0.024
	Verbal fluency	0.432^※※^	0.024	0.156	0.051	−0.035	0.049	−0.395^※^	−0.082	−0.004	−0.277	−0.049	0.002
	Attention	0.014	−0.061	0.066	0.235	−0.006	0.067	0.144	−0.023	0.052	−0.133	−0.033	0.012
	Executive function	0.015	−0.149	0.166	0.287^※^	0.016	0.288	−0.024	−0.100	0.086	−0.183	−0.142	0.023
PANSS													
	Negative	−0.252	−0.237	0.039	−0.070	−0.100	0.060	0.087	−0.063	0.099	0.105	−0.030	0.067
	Positive	0.021	−0.137	0.155	−0.207	−0.185	0.053	0.014	−0.083	0.089	0.183	−0.036	0.108
	General	0.030	−0.110	0.128	0.025	−0.087	0.098	0.079	−0.058	0.082	0.053	−0.049	0.063
Age (years)		−0.011	−0.073	0.067	0.236	−0.005	0.089	0.091	−0.027	0.056	−0.147	−0.045	0.012
JART		−0.068	−0.072	0.044	−0.098	−0.053	0.028	0.221	−0.010	0.058	0.002	−0.025	0.025
*R* ^2^		0.247			0.061			0.067			0.091		

*β-values are standardized values.*

*BACS, Brief Assessment of Cognition in Schizophrenia; PANSS, Positive and Negative Syndrome Scale; JART, Japanese Adult Reading Test.*

*^※^p < 0.05, ^※※^p < 0.01.*

## Discussion

Our examination of sex differences in the social cognitive subdomains of ToM and hostility bias revealed no such differences in patients with early course schizophrenia. Previous studies on sex differences in ToM in schizophrenia reported either no sex differences in ToM (Ayesa-Arriola et al., 2014; Navarra-Ventura et al., 2018, 2021; Riecher-Rossler et al., 2018) or better ToM in females (Abu-Akel and Bo, 2013). This inconsistency in findings might be related to differences in disease duration between the studies. To address this, in the present study, we examined patients with schizophrenia within 5 years of onset. Our study also had no significant sex differences in sociodemographic variables and thus might provide more accurate results for examining sex differences. Although no previous studies have compared sex differences in hostility bias, one study examining whether sex affects hostility bias (Pinkham et al., 2017) had a finding consistent with ours, namely, that sex does not affect hostility bias. We found that there were no sex differences in ToM and hostility bias. In contrast, healthy individuals in previous studies have shown sex differences. These differences can be explained by the effect of estrogen. In the healthy individuals, females have higher ToM (Baron-Cohen et al., 2005; Navarra-Ventura et al., 2021) and lower hostility bias (Baron-Cohen, 2004; Combs et al., 2007; Runions and Keating, 2007). Lower estrogen is associated with lower ToM (Oldershaw et al., 2010; Abu-Akel and Bo, 2013) and higher Hostility (Riecher-Rossler and Kulkarni, 2011). Gonadal hormone levels including estrogen is known to be lower in schizophrenia in 92% of females and 27.7% of males (Howes et al., 2007). Since the decrease in estrogen is greater in females, it is possible that the sex difference observed in the healthy individuals are not present in schizophrenia. Therefore, it was suggested that treatment with estrogen may be effective against ToM and hostility bias.

Interestingly, we found that verbal fluency contributed significantly to ToM in females (*p* < 0.01), whereas executive function contributed significantly to ToM in males (*p* < 0.05). This is the first study to examine the association between ToM and neurocognition in schizophrenia according to sex. To our knowledge, only one study has examined the association between ToM and brain function in schizophrenia by sex (Walsh-Messinger et al., 2019). ToM in females with schizophrenia was found to be associated with the limbic system, whereas ToM in males with schizophrenia was associated with the frontal lobe. In people with schizophrenia and healthy controls, previous studies have shown an association between verbal fluency and the limbic system and between executive function and the frontal lobe (Pihlajamaki et al., 2000; Zhu et al., 2010; Morimoto et al., 2018). Together, the results of our study and previous studies indicate that limbic-based verbal fluency is an important predictor of ToM in females and frontal-based executive function in males. These implications are useful in planning treatment for cognitive rehabilitation. Cognitive rehabilitation aimed at improving ToM should account for deficits in executive function in males and verbal fluency in females. In some cases, it might be helpful to provide treatment for executive function and verbal fluency skills before treatment for ToM.

For hostility bias, higher verbal fluency was significantly associated with lower hostility bias in females (*p* < 0.05), whereas none of the neurocognitive subdomains was significantly associated with hostility bias in males, although verbal fluency may have a marked contribution (*p* = 0.068). No sex differences were found in the association between neurocognition and hostility bias. As far as we know, no previous studies have examined the association between neurocognition and hostility bias in schizophrenia in terms of sex differences, although some studies have consistently reported that the neurocognitive subdomains that contribute to attributional bias are verbal-related ones, such as verbal fluency (Lo and Siu, 2017) and verbal IQ (Donohoe et al., 2008; Buck et al., 2017). Our results also showed that verbal-related neurocognitive subdomains tended to be associated with hostility bias. Previous studies examining the neural basis of hostility in schizophrenia have pointed out an association between hostility and the limbic system (Hoptman et al., 2010; Del Bene et al., 2016; Perlini et al., 2018). As mentioned above, an association also exists between verbal fluency and the limbic system. In fact, hostility and verbal fluency in schizophrenia have a common neural basis, which is in line with our results. Before conducting this study, we expected to find sex differences in the association between neurocognitive subdomains and hostility bias, but we found no such differences. Therefore, female and male patients alike might benefit from similar cognitive rehabilitation strategies to treat hostility bias. In addition to the lack of sex differences in ToM and hostility bias observed in schizophrenia as mentioned above, the associations between neurocognition and ToM and neurocognition and hostility bias also suggest that treatment with estrogen may benefit ToM and hostility bias in both females and males. Previous studies have found that estrogen modifies frontal lobe and limbic system activity during emotion perception tasks, a subdomain of social cognition (Ji et al., 2016; Javed and Charles, 2018). Estrogen is also associated with frontal-based executive function and limbic-based verbal fluency (Ko et al., 2006). Estrogen not only improves frontal-based executive function and limbic-based verbal fluency, but may also be effective in treating ToM and hostility bias, as well as emotion perception. Combining estrogen with neurocognitive and social cognitive rehabilitation may facilitate the treatment of ToM and hostility bias. Further studies focusing on sex differences in hormones might be worthwhile to improve the rehabilitation on social cognition (Sumiyoshi et al., 1997; Gonzalez-Rodriguez et al., 2020). Furthermore, female patients are said to have better prognosis. Our hope is that studying sex differences will uncover critical elements of good outcome thus leading to interventions that will benefit both females and males (Seeman, 2019). By considering associations with cognitive function, it may be possible to provide interventions that take account of sex differences in prognosis.

The limitations of this study are as follows. First, as this was not a longitudinal study, it cannot establish causality. Second, antipsychotic medications that our patients were taking might affect cognitive function; however, the recent literature generally does not report any significant effects of antipsychotics on social cognition (Hempel et al., 2010; Catalan et al., 2018). Third, we assessed ToM and hostility bias using the SCSQ, but it would have been beneficial to have examined other subdomains of social cognition as well. Fourth, in this study, the social cognitive assessment relies on SCSQ which is used as a screening tool. However, ToM and hostility bias domains scores of SCSQ were well-correlated with those of standard measures such as the Hinting task and AIHQ (*r* = 0.52, *P* = 0.0001 for ToM and *r* = 0.34, *P* = 0.05 for hostility bias) (Kanie et al., 2014). We therefore do not believe this undermines the validity of our results. To date, there is no consensus on what kind of scale is appropriate for measuring social cognitive function among the Japanese, and we are currently conducting research to clarify this (Kubota et al., 2021; Okano et al., 2021). We believe it is desirable to use tests that have reached consensus on this. Lastly, this study did not have a healthy control group to compare our results with. Therefore, it is difficult to state whether these results are specific to schizophrenia or reflect the sex differences found in the general population. In order to link our findings to rehabilitation, the differences need to be fully clarified.

## Conclusion

In conclusion, we found sex differences in the associations between neurocognition and ToM and neurocognition and hostility bias in early course schizophrenia, despite the absence of sex differences in ToM and hostility bias. These results suggest that sex differences in neurocognition related to social cognition need to be taken into account to treat social cognition more effectively. These results can be adapted to patients with early course schizophrenia and contribute to the promotion of future treatments targeting social cognition.

## Data Availability Statement

The datasets presented in this article are not readily available because participants of this study did not agree for their data to be shared publicly. Requests to access the datasets should be directed to RK, ryo-okubo@ncnp.go.jp.

## Ethics Statement

The studies involving human participants were reviewed and approved by the Institutional Review Board of the National Center of Neurology and Psychiatry. Written informed consent to participate in this study was provided by the participants’ legal guardian/next of kin.

## Author Contributions

RK, RO, SI, and NY contributed to writing—original draft preparation. RO, SI, and NY contributed to supervision. All authors contributed to conceptualization and writing—review and editing, read and agreed to the published version of the manuscript.

## Conflict of Interest

The authors declare that the research was conducted in the absence of any commercial or financial relationships that could be construed as a potential conflict of interest.

## Publisher’s Note

All claims expressed in this article are solely those of the authors and do not necessarily represent those of their affiliated organizations, or those of the publisher, the editors and the reviewers. Any product that may be evaluated in this article, or claim that may be made by its manufacturer, is not guaranteed or endorsed by the publisher.
